# Tree resilience to drought increases in the Tibetan Plateau

**DOI:** 10.1111/gcb.14470

**Published:** 2018-10-29

**Authors:** Ouya Fang, Qi‐Bin Zhang

**Affiliations:** ^1^ State Key Laboratory of Vegetation and Environmental Change Institute of Botany Chinese Academy of Sciences Beijing China

**Keywords:** climate change, extreme drought, recovery, resistance, spatial resilience, tree rings

## Abstract

Forests in the Tibetan Plateau are thought to be vulnerable to climate extremes, yet they also tend to exhibit resilience contributing to the maintenance of ecosystem services in and beyond the plateau. So far the spatiotemporal pattern in tree resilience in the Tibetan Plateau remains largely unquantified and the influence of specific factors on the resilience is poorly understood. Here, we study ring‐width data from 849 trees at 28 sites in the Tibetan Plateau with the aim to quantify tree resilience and determine their diving forces. Three extreme drought events in years 1969, 1979, and 1995 are detected from metrological records. Regional tree resistance to the three extreme droughts shows a decreasing trend with the proportion of trees having high resistance ranging from 71.9%, 55.2%, to 39.7%. Regional tree recovery is increasing with the proportion of trees having high recovery ranging from 28.3%, 52.2%, to 64.2%. The area with high resistance is contracting and that of high recovery is expanding. The spatiotemporal resistance and recovery are associated with moisture availability and diurnal temperature range, respectively. In addition, they are both associated with forest internal factor represented by growth consistence among trees. We conclude that juniper trees in the Tibetan Plateau have increased resilience to extreme droughts in the study period. We highlight pervasive resilience in juniper trees. The results have implications for predicting tree resilience and identifying areas vulnerable to future climate extremes.

## Graphical abstract

Tree resilience is important for maintaining the healthy growth of forests, yet remains largely unquantified. Examination of tree resilience to drought extremes from 28 sites of juniper forests in the Tibetan Plateau reveals a temporal decreasing trend in tree resistance and an increasing trend in recovery, and a spatial contraction in the area of high resistance and expansion in high recovery. The variation of tree resilience is associated with moisture availability, diurnal temperature range, and growth consistence among trees. The results have implications for predicting tree resilience and identifying areas vulnerable to future climate extremes.

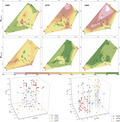

## 1 INTRODUCTION

Extreme drought events have had serious impacts on tree growth in recent decades (Ciais et al., 2005; Trumbore, Brando, & Hartmann, 2015) and are expected to have even more severe effects with climate warming (Trenberth et al., 2014). Widespread reductions of tree growth and increased forest mortality triggered by warming‐induced droughts have been reported worldwide (Gentilesca, Camarero, Colangelo, Nole, & Ripullone, 2017; O'Brien, Leuzinger, Philipson, Tay, & Hector, 2014). However, trees in forests do not merely passively respond to droughts but develop eco‐physiological resilience to resist the influence and to recover from droughts (Holling, 1973; Thompson, Mackey, Mcnulty, & Mosseler, 2009). Tree resilience to climate extremes has risen to the forefront of planning sustainable forestry, but is difficult to quantify, particularly in heterogeneous landscapes (Allen et al., 2016).

Spatial heterogeneity of habitats in forested landscapes results in differences in resilience among tree stands. In this context, characterization of spatial pattern in resilience is prerequisite for explanation and understanding of the driving forces responsible. For example, pine trees along a rainfall gradient in the Eastern Mediterranean showed higher resilience to drought through eco‐physiological adjustments in sustaining trees at dry conditions than humid conditions (Helman, Lensky, Yakir, & Osem, 2017). In the Amazon rainforest, tree resilience to climate extremes is lower in floodplains than uplands, suggesting that vulnerability to wildfires is responsible for the pattern of spatial resilience (Flores et al., 2017). On the other hand, the regional tree resilience may not be a static spatial pattern, but it changes under the influence of climate change. Forested landscape in north‐central Minnesota showed that climate change would reduce resilience (Lucash, Scheller, Gustafson, & Sturtevant, 2017). In northeastern Spain, tree resilience showed a progressive reduction over time, which could be mostly influenced by tree size and drought intensity (Serra‐Maluquer, Mencuccini, & Martinez‐Vilalta, 2018). Further understanding of spatial resilience in temporal dimension is limited by availability of long and high‐resolution data.

Tree rings provide long and annual resolution record of growth response to climate, resulting highly interest for the study of extreme events. Trees sensitive to moisture often reduce their radial growth if drought occurs, and recover the growth as to predrought conditions, when rain subsequently increases. The tree response to drought is an eco‐physiological process, allowing different growth performance in individual trees depending on their extrinsic (e.g., habitat and competition) and intrinsic (e.g., health and genetic) factors (Willis, Jeffers, & Tovar, 2018). Embedded in this process is tree resilience which could be evaluated by comparing ring‐widths prior to, during, and after drought events. Lloret, Keeling, and Sala (2011) proposed indices to measure tree resilience, resistance, and recovery, using ring‐width data from individual trees, and reported that tree resilience to recent events is not significantly different from that in the past. Using similar approach of resilience calculation, other researchers found that tree resilience to drought events is not stable in time and space (Cole, Bhagwat, & Willis, 2014; Gazol et al., 2018; Xu et al., 2016). Most studies of forest resilience are based on examination of tree‐ring chronologies. For example, Vitali, Buntgen, and Bauhus (2017) used 18 tree‐ring chronologies to assess forest resilience of three species at different altitudes in Germany. Gazol, Camarero, Anderegg, and Vicente‐Serrano (2017) used 775 tree‐ring chronologies of Northern Hemisphere to explore the impact of drought on forest resilience. These tree‐ring chronologies are the mean growth of trees in the forest, with no regard to difference among individual trees. To date, it remains unclear how the spatial tree resilience changes in historical disturbances. Lack of coupled spatiotemporal data is main obstacles preventing comprehensive understanding of the spatial resilience, particularly for heterogeneous forested landscapes.

The Tibetan Plateau, known as the “Third Pole” of the earth, is characterized by heterogeneous landscapes, having a large number of high mountains, lakes, and rivers, and experiencing greater rate of recent warming than elsewhere of the same latitude (Liu & Chen, 2015). Growth of trees in the Tibetan Plateau is slow in the severe alpine climate but highly sensitive to climate change and extremes (Shao et al., 2009; Zhang, Evans, & Lyu, 2015). Forests in the Tibetan Plateau are distributed in heterogeneous landscapes with little human activities. They play an important role in maintaining ecosystem services in and beyond the plateau (Wang, Li, Ren, & Liu, 2007). However, little attention has been paid to tree resilience in this region, despite extensive studies of climate change in the Tibetan Plateau (Bräuning, Grießinger, Hochreuther, & Wernicke, 2016). In this study, we address the spatial resilience to droughts using our accumulation of tree‐ring data across the forests in the Tibetan Plateau. We consider the tree resilience as the combination of tree resistance and recovery, which means the ability of tree to return to the conditions following the perturbation. The study aimed to answer two questions: (a) How does the spatial resilience change over time? and (b) What determines the variation of tree resilience?

## 2 MATERIALS AND METHODS

### 2.1 Tree‐ring data

**Figure 1:**
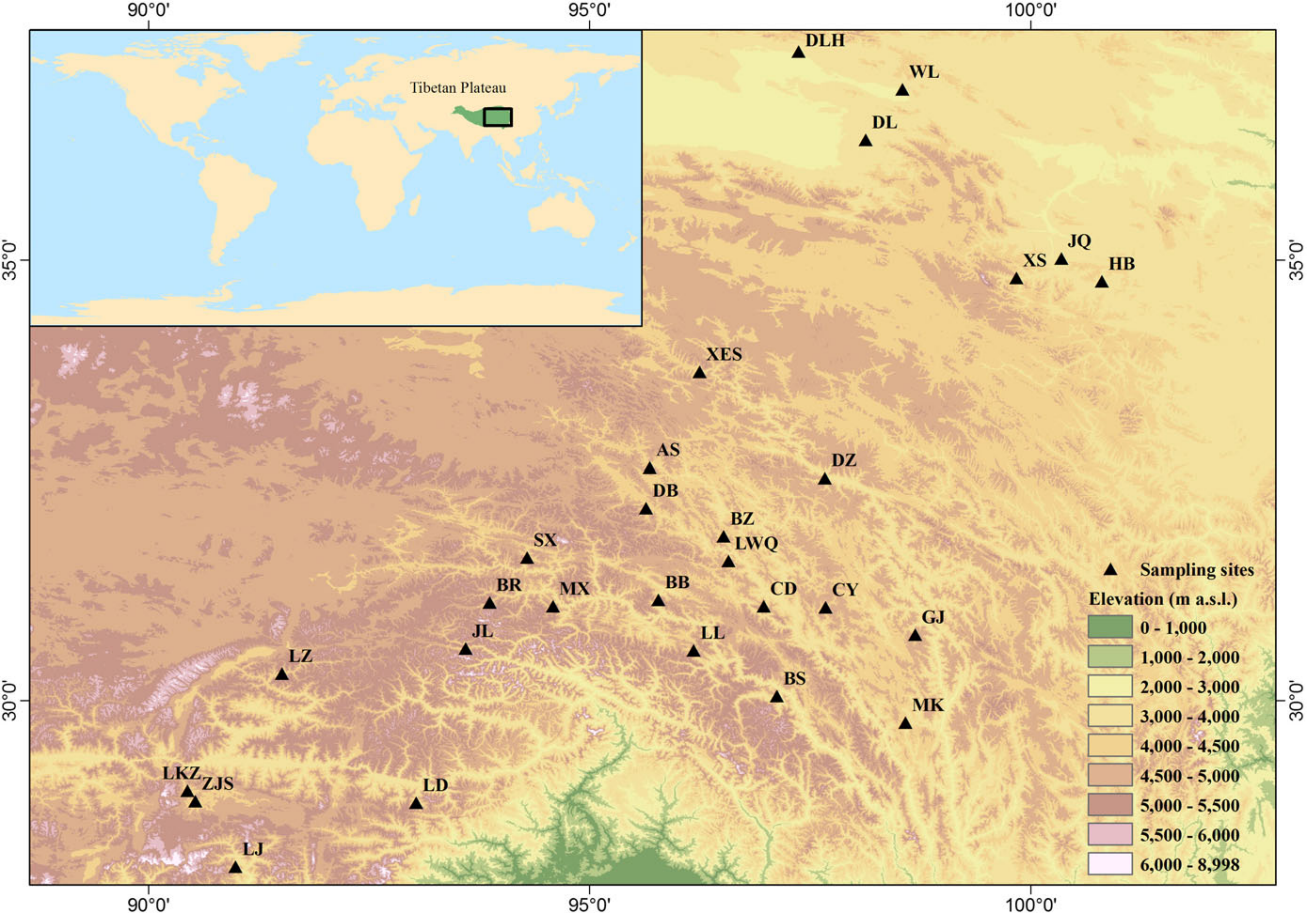
Location of the study region. Tree‐ring sampling sites are indicated by black triangles. The full names of site abbreviations are listed in Supporting Information Table S1

Increment cores were collected from 849 trees in 28 juniper forests (*Juniperus* spp.) in the Tibetan Plateau over a geographic range from 28°7′ to 37°23′N and from 90°26′ to 100°49′E and with an elevation ranging from 3,300 to 4,700 m a.s.l. (Figure 1, Supporting Information Table S1). Forests with dominant species of *Juniperus prezwalskii* and *J. tibetica* occur in the northern and southern part of the Tibetan Plateau, respectively. Twenty tree‐ring site chronologies were previously described (Fang, Alfaro, & Zhang, 2018; Zhang et al., 2015) and eight site chronologies were newly reported here. All the sampled increment cores were dried and mounted on grooved sticks and polished using increasingly fine sanding paper to expose tree‐ring details to cellular level. Tree‐ring widths were measured to an accuracy of 0.001 mm using a LINTAB 6 system (Frank Rinntech, Heidelberg, Germany). Tree rings were crossdated by standard dendrochronological techniques (Schweingruber, 1988) and the quality of crossdating was validated using the COFECHA program (Holmes, 1983).

For each individual tree, the growth trend due to nonclimatic effects was removed using a cubic spline of 50% frequency‐response cutoff at half length of the series (Cook, Briffa, Shiyatov & Mazepa, 1990). The resultant ring‐width indices of all trees form the dataset for calculation of tree resistance and recovery. Biweight robust mean of the ring‐width indices for each site was computed to develop the site tree‐ring chronology.

### 2.2 Extreme drought events

The detection of extreme drought events is based on self‐calibrating Palmer Drought Severity Indices (scPDSI) (Schrier, Barichivich, Briffa, & Jones, 2013) spanning from 1957 to 2000 and covering the region from 27°45′ to 37°45′N and from 90°15′ to 101°15′E (Supporting Information Figure S1). The scPDSI is an improved index which represent soil moisture condition with consideration of atmospheric input and soil evaporation (Wells, Goddard, & Hayes, 2010). Trees do not grow during the ground‐frozen period from approximately mid‐October to early April (Kang & Zhang, 2001; Zhao et al., 2004). We used the data of averaged May–June scPDSI to characterize the drought extremes. The first order differences in these data were calculated and the values that are 1.5 times standard deviation below the mean occurred in years 1969, 1979, and 1995 (Supporting Information Figure S2). These 3 years are considered as extreme drought events that will be used in the analysis of tree resilience to droughts.

### 2.3 Indices of tree resistance and recovery

We considered that the tree resilience is reflected by trees’ resistance to perturbation and their ability to recover to the original conditions. The high resilience corresponds to low resistance and high recovery. The two components of tree resilience, tree resistance (Rt) and tree recovery (Rc), were calculated for individual trees following the formulas proposed by Lloret et al. (2011).

(1) Rt=Dr/PreDr

(2) Rc=PostDr/Dr

where, Dr indicates the ring‐width index in the year of drought; and PreDr and PostDr indicate the mean ring‐width indices during the 4 years before and after the drought. Calculations were executed using the R package “pointRes” (Maaten‐Theunissen, Maaten, & Bouriaud, 2015).

Indices of tree resistance and recovery to the drought events in years 1969, 1979, and 1995 were calculated for each tree at each site. We consider a tree as high resistance if its Rt is >0.75, and a tree as high recovery if its Rc > 1.25. The proportion of trees having high resistance (P_Rt > 0.75_) to the drought events was computed for each site, and so did for the proportion of trees having high recovery (P_Rc > 1.25_).

### 2.4 Spatiotemporal characteristics of tree resistance and recovery

Spatial features of tree resistance and recovery in extreme drought events were analyzed using spatial interpolation in the Spatial Analyst tool in ArcGIS 10.3 software. The closest subsets of P_Rt > 0.75_ and P_Rc > 1.25_ values were integrated to create the Thiessen polygons, respectively. Growth performance of trees in response to the three drought events was compared to obtain the temporal features of tree resistance and recovery.

### 2.5 Analysis of influencing factors

Climatic and forest internal factors were examined to detect the driving forces of the spatiotemporal pattern of tree resistance and recovery. The climatic factors in analysis include temperature (monthly mean temperature, monthly maximum temperature and monthly minimum temperature), precipitation, scPDSI, and diurnal temperature range (DTR). These data were obtained from the Climatic Research Unit (CRU) 4.01 dataset (http://climexp.knmi.nl) since the year 1957. Climate in the sampling sites was represented by the nearest grid‐point values. Forest internal factors include mean age of trees in each site, standard deviation of tree‐ring indices, and consistence of growth among trees in 11‐year period (including the event year and 5 years before and after the event). The consistence of growth was represented by GLK (Gleichläufigkeit) index (Schweingruber, 1988), which measures the coherence of year‐to‐year growth change between two trees using the R package “dplR” (Bunn, 2008).

Stepwise regression models were employed to establish the relationship between driving factors and the tree resistance and recovery. Least squares method was applied to determine the degree of reliability of the models. Independent variables are entered if their *p*‐values < 0.1 and are removed if their *p*‐values are >0.15.

## 3 RESULTS

### 3.1 Tree growth and spatial resilience

Radial growth of juniper trees in the 28 sampling sites was positively correlated with scPDSI in May‐June with an average correlation coefficient of 0.47 in the interval 1957–2000 (Supporting Information Table S1). The following analysis is based on tree‐ring indices of individual trees from these 28 sites.

The mean of tree‐ring indices in drought years is lower than in years before and after the drought event (Supporting Information Figure S3). Missing rings occurred at a relatively high rate in the three drought extreme years although they also occurred in other years (Supporting Information Figure S4). The ratio of mean tree‐ring indices in 1–5 years before the drought year and that of the event year is >1, and so do those 1–5 years after the event. These observations indicate that trees reduced their radial growth in the three drought years and rapidly recovered afterward.

**Figure 2:**
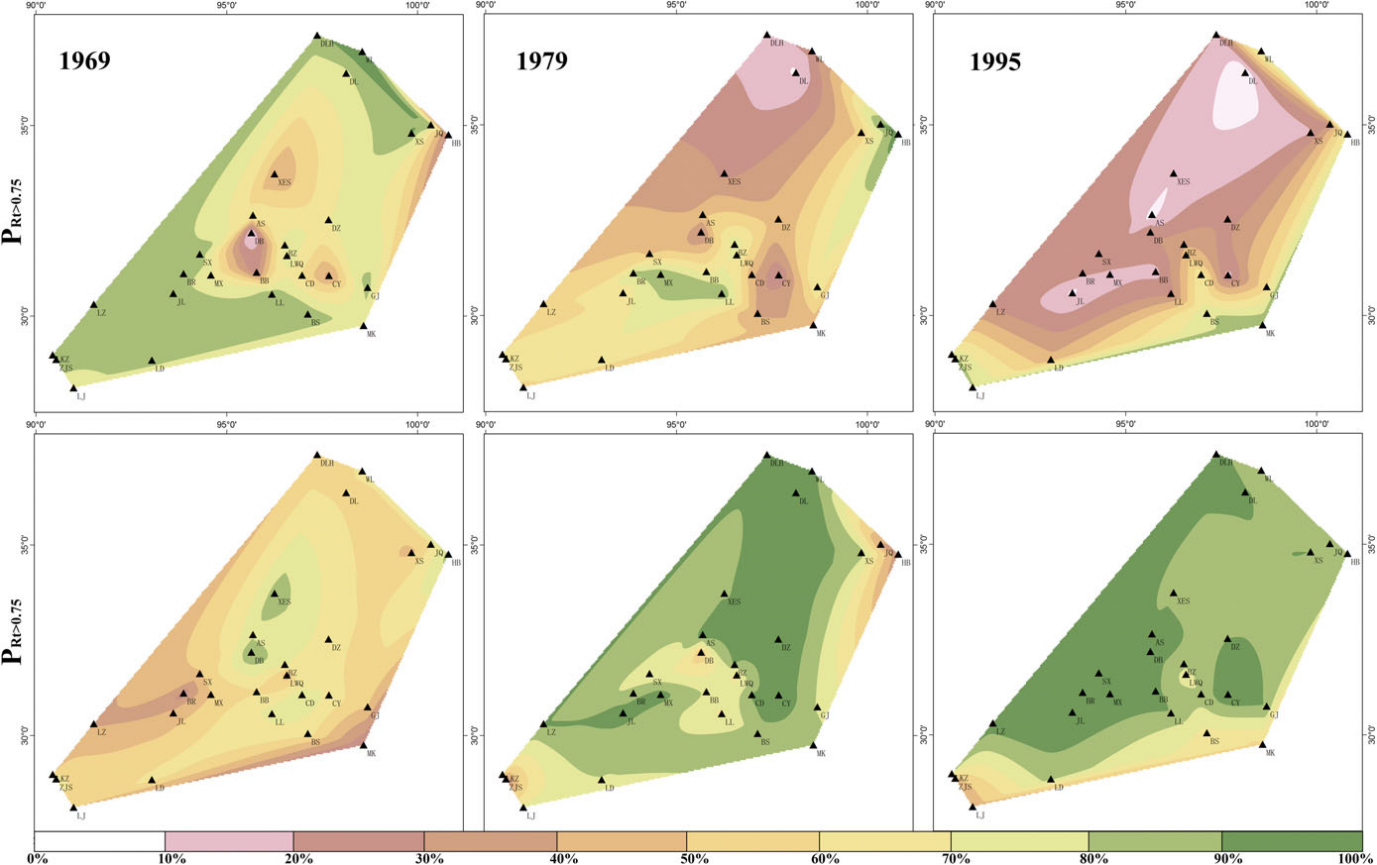
Spatial patterns of regional tree resistance (Rt) and recovery (Rc) in the drought years of 1969, 1979, and 1995. Colors represent the percentage of trees having high resistance (P_Rt > 0.75_) and high recovery (P_Rc > 1.25_)

When mapping the regional tree resistance and recovery of the 28 sites by values of percentage of trees having high resistance (P_Rt > 0.75_) and high recovery (P_Rc > 1.25_), the spatial resilience showed different patterns in the three drought events (Figure 2). Area of high resistance is contracting and that of high recovery is expanding. The low‐resistance center is moving north. In 1969, trees near the center of the study region had low resistance, especially in DB site where only 4.2% trees had high resistance. In 1979 and 1995, trees in most areas of the northern part had relatively low resistance with the lowest value occurring in DL site. Conversely, a small area of tree stands in the central region had high recovery in 1969 and larger area of tree stands exhibited high recovery in 1979 and 1995. The high‐recovery center is swinging in the heart of the study area.

### 3.2 Change in tree resilience over time

**Figure 3:**
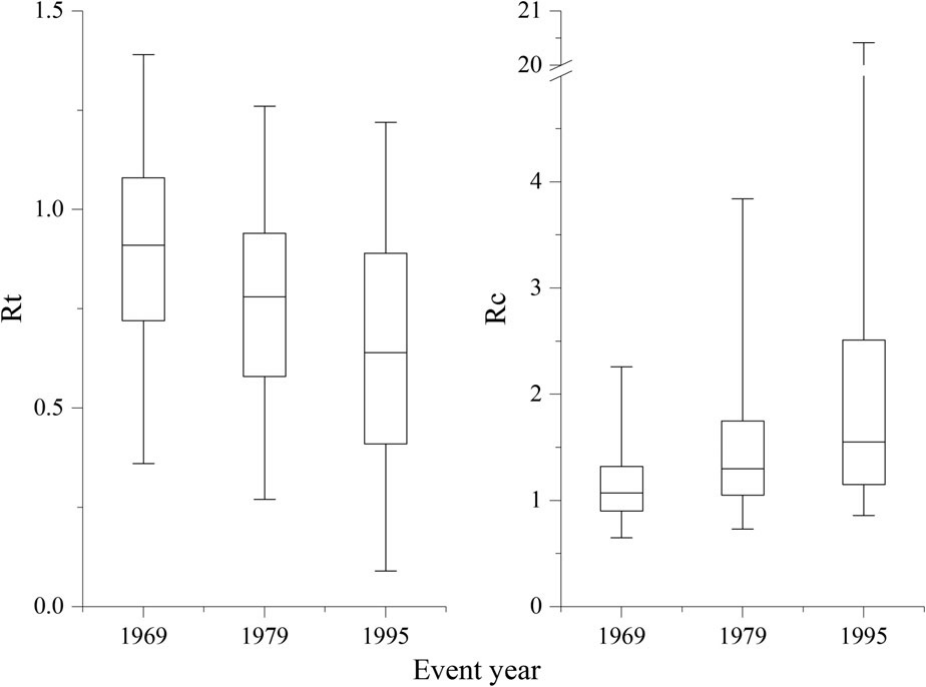
Boxplots of resistance (Rt) and recovery (Rc) for individual trees in the three drought events. The boxes indicate 25th and 75th quartiles, and the lines in the middle of the box indicate the medium values. Significance (*p* < 0.01) of the difference between groups in the three events was verified using Kruskal–Wallis test

In temporal dimension, the tree resistance showed a decreasing trend from drought years in 1969 to 1979 and 1995, whereas the tree recovery showed an increasing trend (Figure 3). The mean resistance of 849 trees in the 28 sites was 0.90, 0.78, and 0.65 to the drought years in 1969, 1979, and 1995, respectively. The proportion of trees having high resistance to the three drought years was 71.9%, 55.2%, and 39.7%, respectively (Supporting Information Figure S5). The mean of tree recovery to the three drought years was 1.31, 1.69, and 10.03 in 1969, 1979, and 1995, respectively. The proportion of trees having high recovery following the three drought years was 28.3%, 52.2%, and 64.2%, respectively (Supporting Information Figure S6).

### 3.3 Effect of external and internal factors on tree resilience

**Figure 4:**
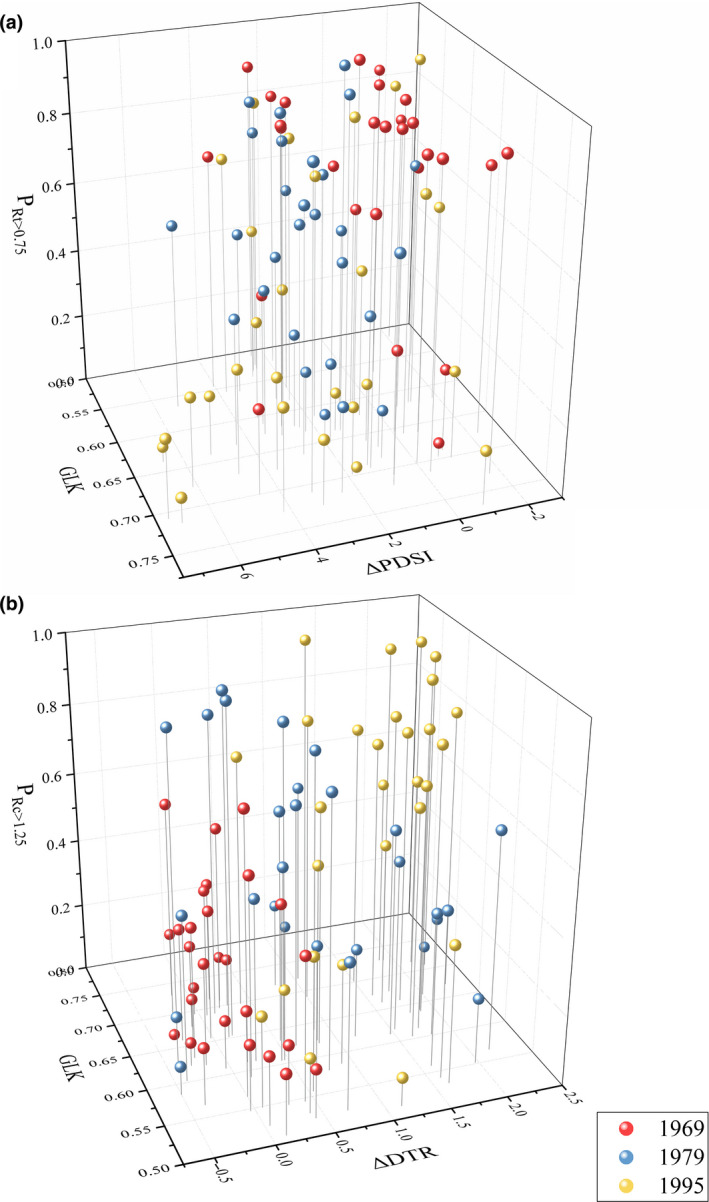
Scatterplots of regional tree resistance and recovery with their predicting factors. Proportion of trees having high resistance in relation to May‐June ΔPDSI (scPDSI difference between the drought year and the year before the drought) and the GLK values (11‐year period including the event year and 5 years before and after the event) among individual trees (a), and proportion of trees having high recovery in relation to May–July ΔDTR (DTR difference between the year after the drought and the drought year) and the GLK values (11‐year period including the event year and 5 years before and after the event) among individual trees (b)

Stepwise regression model showed that the P_Rt > 0.75_ values from the 28 sites in the three drought events were negatively associated with May–June ΔPDSI (difference of scPDSI between the drought year and the year before) and GLK (Equation 3, Figure 4). The P_Rc > 1.25_ values were positively associated with May–July ΔDTR (difference of DTR between the year after the drought and the drought year) and GLK (Equation 4, Figure 4).

(3) $$\begin{array}{cc} {\text{P}}_{\mathrm{Rt}>0.75}= & 1.887-0.052\;\Delta \text{PDSI}-1.97\;\text{GLK}\\ & \text{Statistic}:\;p<0.001,\;R^{2}=0.35 \end{array}$$

(4) $$\begin{array}{cc} {\text{P}}_{\mathrm{Rc}>1.25}= & -0.5413+0.154\;\Delta \text{DTR}+1.45\;\text{GLK}\\ & \text{Statistic}:\;p<0.001,\;R^{2}=0.32 \end{array}$$

## 4 DISCUSSION

### 4.1 Quantification of tree resilience to drought

We quantified the spatial resilience to three extreme droughts by calculating tree resistance and recovery and the proportions of trees having high resistance and recovery in 28 sites in the Tibetan Plateau. These multiple‐scale data provide fundamental insights into the pattern of changing spatial resilience and interaction of external and internal factors.

We consider that the regional tree resilience could be better reflected from the growth of individual trees than the mean growth of trees. Trees in the forest often show different strategies to cope with extreme events. For example, trees of different size or health status may respond differently to climate extremes (Serra‐Maluquer et al., 2018); trees in extremely stressful local habitats may have missing rings after a very dry May and June in the Tibetan Plateau (Liang, Leuschner, Dulamsuren, Wagner, & Hauck, 2016). We therefore examined the resistance and recovery from individual trees and further analyzed the proportion of trees having high resistance and recovery. The degree of regional tree resistance could be evaluated from the number of trees having high resistance. A region having high resistance should have many resistant trees, not merely a few high resistant individuals. The use of proportion of trees having high resistance provides a window to look at the performance of individual trees and avoids the effects of extremely high or low values such as those derived from trees having missing rings. The same applies to the regional tree recovery. This new approach addresses the growth difference in individual trees and provides sights into the nature of forest.

### 4.2 Increased tree resilience and its causes

Our results showed that tree resistance is decreasing and tree recovery is increasing (Figure 3), which accompanies a contraction in the area of high resistance and an expansion in the area of high recovery (Figure 2). This observation indicates an increased tree resilience, which is represented by the increased magnitude of tree reaction to drought events. Previous studies addressed that resilience features a regular variation with climatic and geographic conditions. Lucash et al. (2017) reported that climate change lowered forest resilience in their studies of climate projections across north‐central Minnesota. Li, Wu, Liu, Zhang, and Li (2018) found that resilience exhibited an obvious unimodal variation along the gradient of latitude. We hypothesize that the temporal‐spatial tree resilience is influenced by not only climatic factors but also forest internal factors.

We found that moisture availability, as represented by annual change in scPDSI (ΔPDSI), is a factor influencing regional tree resistance. The greater the interannual change in scPDSI, the lower the regional tree resistance. We concurred with other studies (Gazol et al., 2017; van de Koppel & Rietkerk, 2004) that changes in drought intensity are important in influencing the tree resistance. Drought intensity is usually considered the main driver causing growth reduction in trees (Anderegg, 2015). However, trees can develop adaptation to their local habitats, thus not obviously responding to long‐term change but sensitive to sudden change in moisture availability. Chronically stressed trees in dry condition may better survive in drought events, whereas trees growing in normal conditions have less resistance to the same intensity of droughts (McNulty, Boggs, & Sun, 2014).

We found for the first time that annual change in diurnal temperature range is an important factor influencing regional tree recovery. The greater the interannual increase in diurnal temperature range, the higher the ability of tree recovery. In cold area, tree recovery after droughts depends on its ability to initiate cambial growth in spring which is closely linked to temperature regulation (Ford, Harrington, Bansal, Gould, & St. Clair, 2016; Li, Rossi, Liang, & Camarero, 2016). Cloud cover, which largely determine the mean magnitudes of DTR, affects greatly daily maximum temperature and daytime evapotranspiration on plateau (Dai, Trenberth, & Karl, 1999). Changes in DTR influence the process of photosynthesis and the capacity of trees to use the products of photosynthesis in alpine trees, which contribute to regional tree recovery (Pregitzer, King, Burton, & Brown, 2000). It was reported that wide diurnal amplitude in temperature plays an important role in tree growth in arctic and alpine forest (Körner, 1998).

As for forest internal factors, we found that growth consistence among trees, as represented by GLK values, is an important factor influencing both regional tree resistance and recovery. The greater the diverse growth among trees, the higher the tree resistance, and the lower the tree recovery. Similar to the observations that increased biodiversity has positive effect on ecosystem resistance and negative effect on recovery (Sakschewski et al., 2016; Wu et al., 2017), the diverse growth in trees may promote tree resistance by dampening the effect of droughts on the population, but inhibit recovery by diluting the effect of favorable condition after droughts (Isbell et al., 2015; Mori, Furukawa, & Sasaki, 2013; Thompson et al., 2009). After all, the reliability of the tree resilience model (Equations 3 and 4; Figure 4) was improved when both forest external and internal factors are taking into account.

### 4.3 Risk in tree resilience

Our study showed an increasing trend in tree recovery during 1957 to 2000, but the resistance to droughts is decreasing. In space, although the area of high recovery is increasing, the area of low resistance is increasing too (Figure 2), suggesting that the risk of tree decline exists in the region. If the change in moisture availability exceeds the threshold, the trees would face the risk of growth decline because they may not be able to resist drought extremes (Allen et al., 2016; Holling, 1973; Mori et al., 2013). Furthermore, if sustained drought extremes occur, they may synchronize the growth among trees leading to increased risk in tree decline. These potential threats bring uncertainty in assessment of tree resilience because the resilience may change abruptly in gradually changing climate. For example, van de Koppel and Rietkerk (2004) found that plant standing crop suddenly collapses if rainfall is reduced below a threshold. Ponce Campos et al. (2013) showed that the water‐use efficiency of grassland decreased in prolonged drought, resulting in decrease in resilience and reorganization of the composition and structure. The trees in the northern Tibetan Plateau are vulnerable to climate change and experienced a major episode of forest mortality in the late 18th century (Fang et al., 2018).

Our results showed that trees in heterogeneous environment developed different strategies to cope with extreme droughts. The environmental heterogeneity poses greater risks in areas sensitive to drought extremes. Gazol et al. (2018) demonstrated that tree resilience varies across biomes and that trees inhabiting temperate and continental sites might have less ability to recover from intense droughts. Due to the complexity in heterogeneous forest landscapes, it is still hard to answer how tree resilience changes in biome or in the same climate system. We suggest that the risk of resilience in heterogeneous forests in the Tibetan Plateau depends on the changes in moisture, diurnal temperature, and growth consistence among trees.

We conclude a temporal decreasing trend in tree resistance and an increasing trend in recovery, and a spatial contraction in the area of high resistance and expansion in high recovery. The spatial‐temporal change of regional tree resilience is related to growth consistency among trees, moisture, and diurnal temperature conditions in all sampling sites. Assessment of tree resilience in future should take into account both tree resistance and recovery and the factors influencing them.

## CONFLICT OF INTEREST

We declare that there are no conflict of interests.

## AUTHOR CONTRIBUTIONS

O.F. conducted data analysis, prepared the figures, and wrote the manuscript; Q.Z. organized the project, commented on the analysis, and revised the manuscript.
