# Supplementary material for: Tree resilience to drought increases in the Tibetan Plateau
Source: Glob Chang Biol. 2018 Oct 29;25(1):245–53. doi: 10.1111/gcb.14470 (PMC7379736; doi:10.1111/gcb.14470)
Supplement: Supplementary file 1 [file GCB-25-245-s001.docx]

Fig. S1 Mean scPDSI covering the sampling sites in May-June since the year 1957. The red line is the fitted linear regression line.





Fig. S2 First order differences of mean scPDSI covering the sampling sites in May-June since the year 1958. Drought years marked by red points are defined with the value less than mean value minus standard deviation (dashed line).


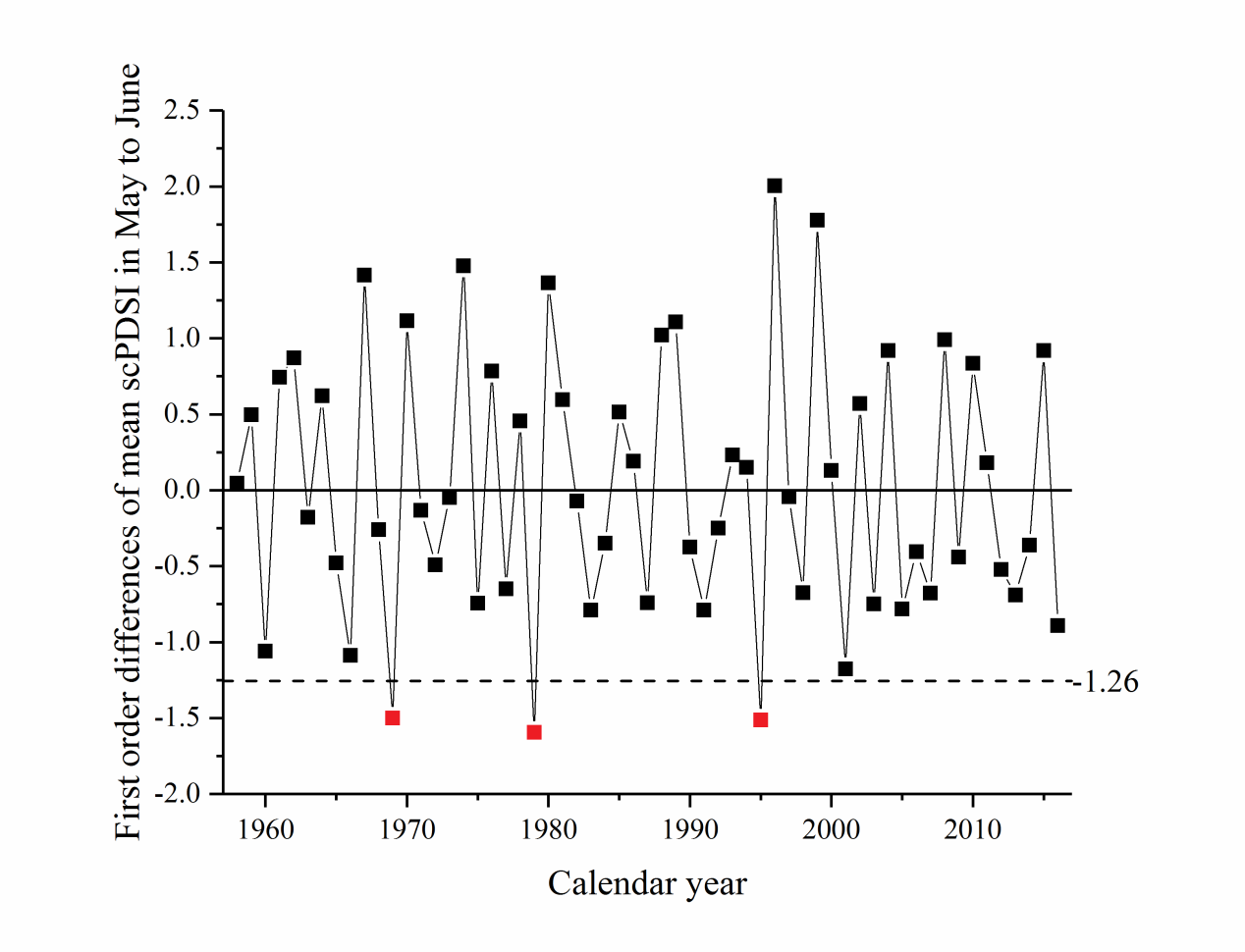


Fig. S3 Tree-ring indices in the three drought year (Dr) and the mean tree-ring indices in the four years before (PreDr) and after (PostDr) the drought years. Thick solid lines in the middle of the box indicate the median values, boxes indicate 25th and 75th quartiles, and the crosses show the mean values.





Fig. S4 Rate of missing rings of all sampled trees. The three drought events are marked with red triangles.


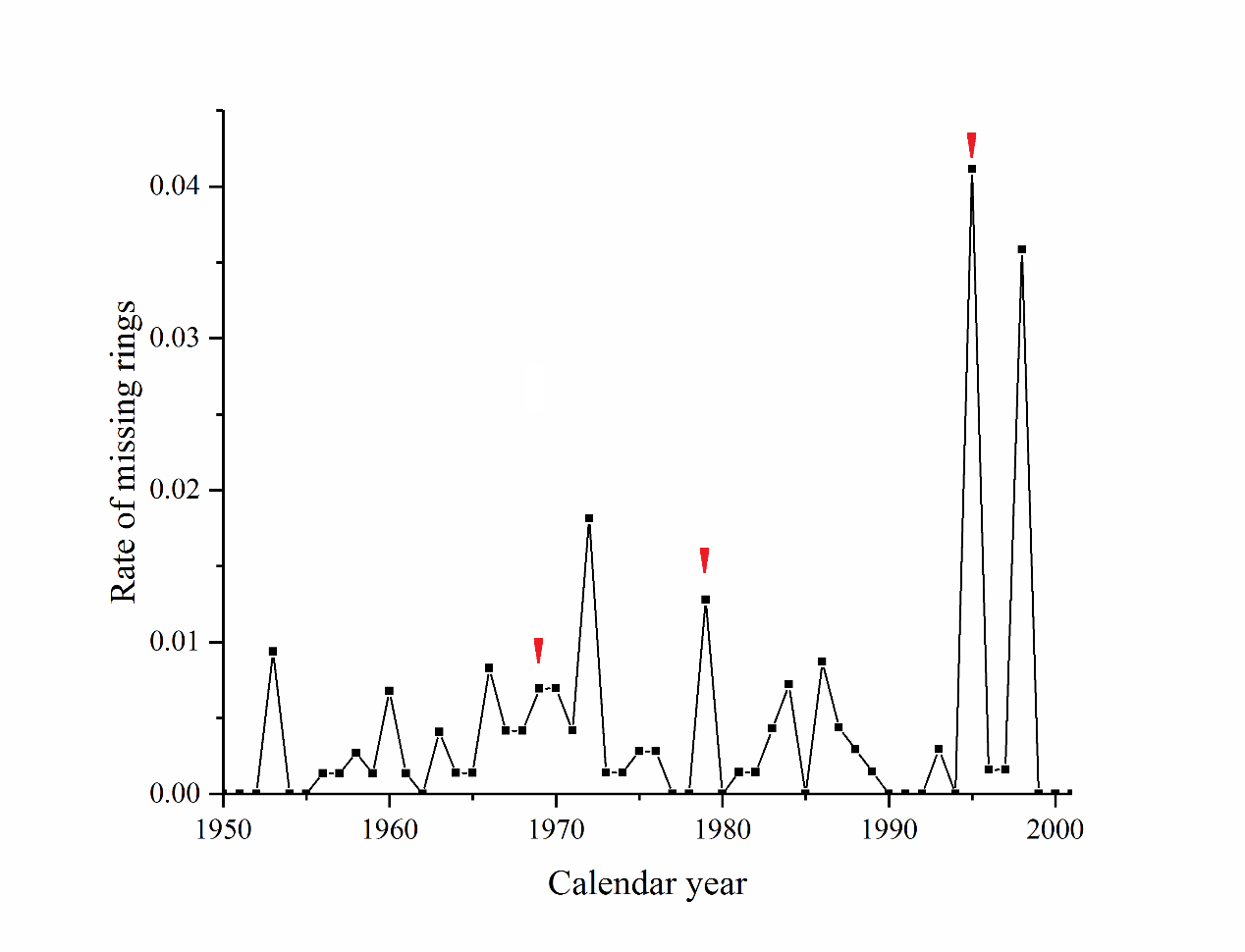


Fig. S5 Proportion of trees having high resistant (P_Rt > 0.75_) in each sampling site in the drought years of 1969, 1979 and 1995 A.D..


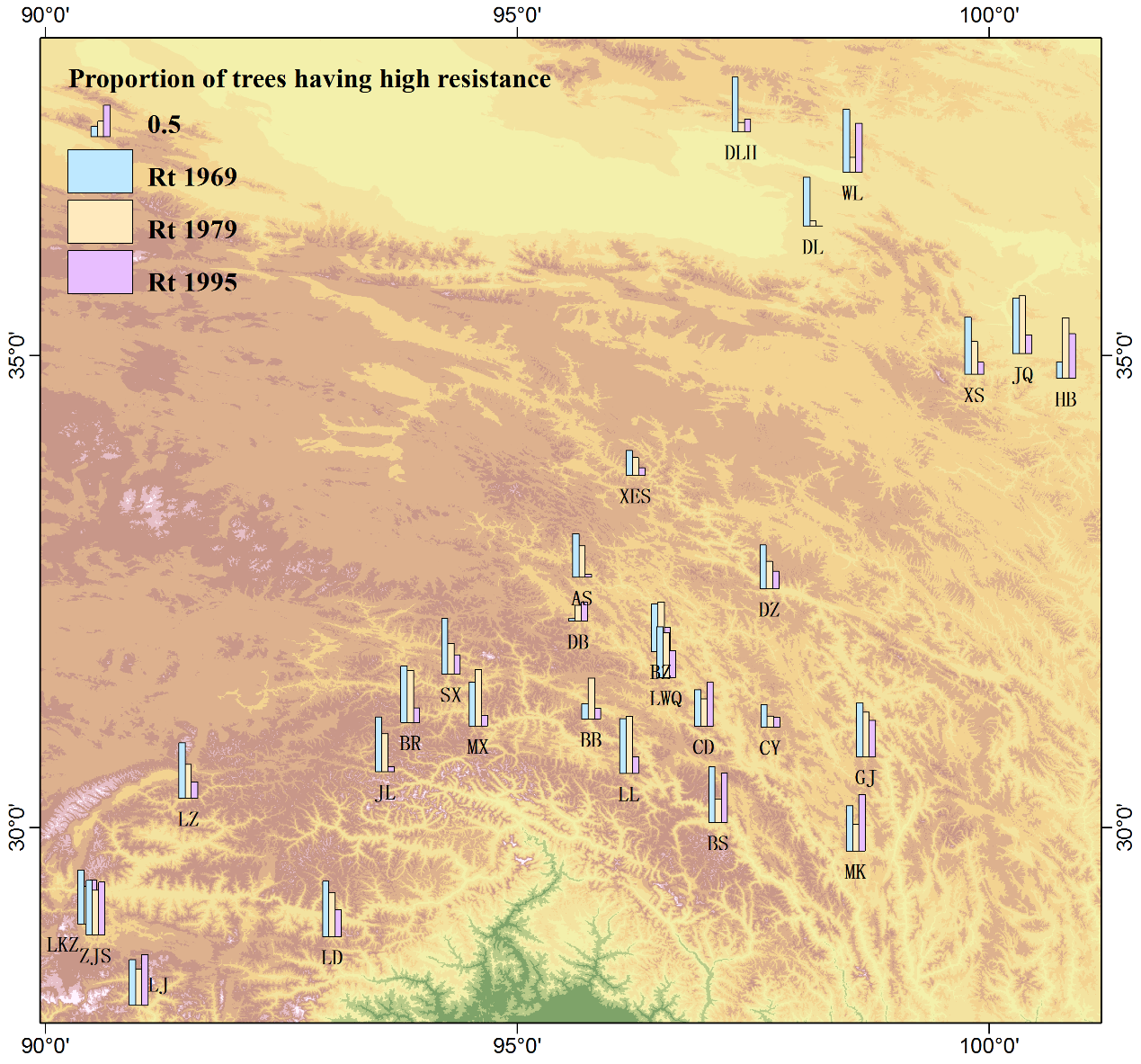


Fig. S6 Proportion of trees having high recovery (P_Rc > 1.25_) in each sampling site in the drought years of 1969, 1979 and 1995 A.D..


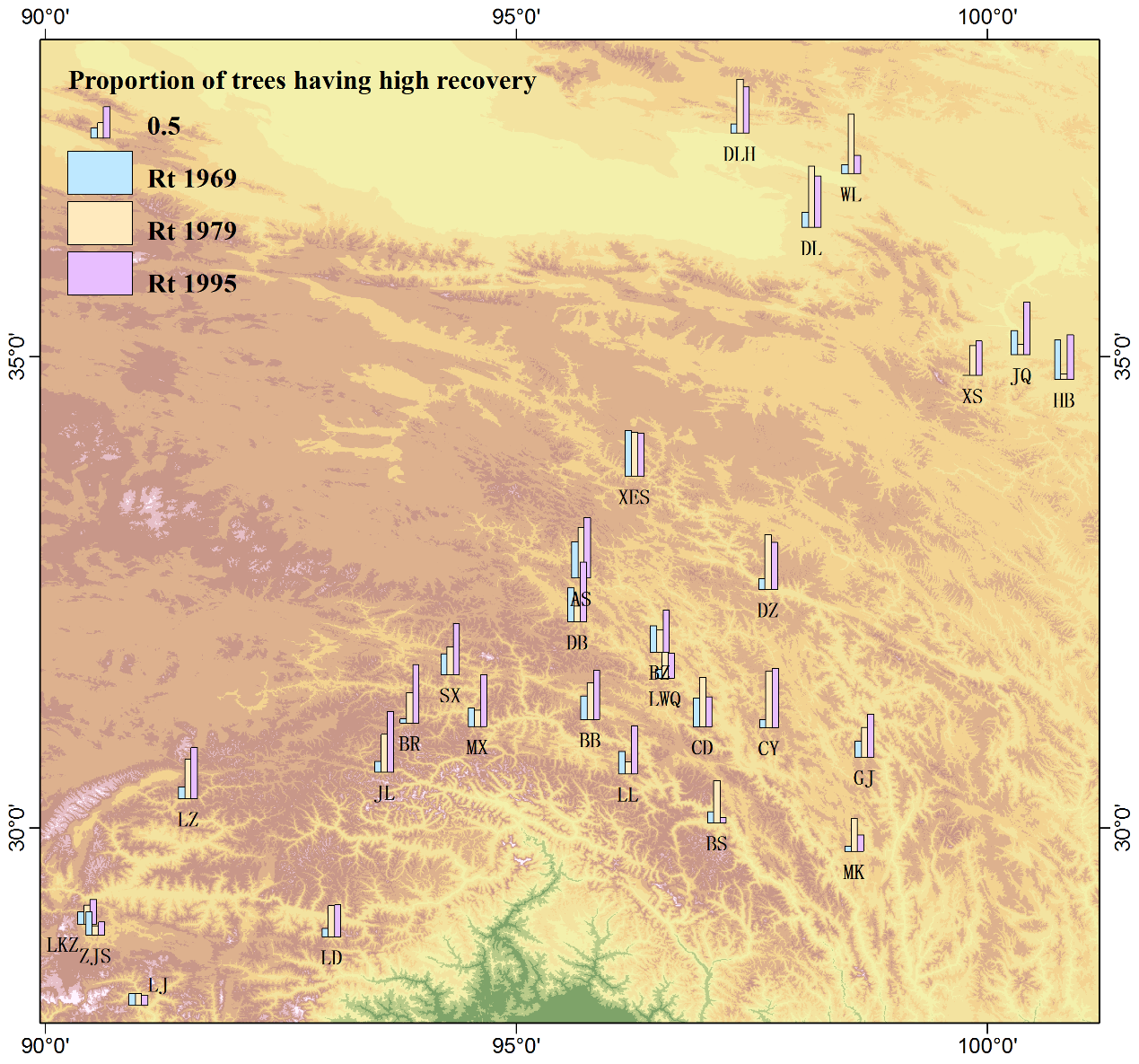


Table. S1 Information about the 28 tree-ring chronologies in the Tibetan plateau.

| Site No. | Site name | Latitude (°N) | Longitude (°E) | Elevation (m a.s.l.) | Mean age of trees (1969) | Period (A.D.) | Number of trees | Mean R_bt_ | R_chron-PDSI_ |
| --- | --- | --- | --- | --- | --- | --- | --- | --- | --- |
| 1 | DLH* | 37.37 | 97.37 | 3820 | 682.2 | 951-2001 | 37 | 0.757 | 0.617 |
| 2 | WL* | 36.94 | 98.55 | 3640 | 453.4 | 1322-2001 | 22 | 0.584 | 0.482 |
| 3 | DL* | 36.37 | 98.13 | 3610 | 637.7 | 157-2000 | 69 | 0.702 | 0.601 |
| 4 | JQ* | 35.02 | 100.35 | 3626 | 368.9 | 1404-2005 | 27 | 0.463 | 0.301 |
| 5 | XS* | 34.80 | 99.84 | 3644 | 449.7 | 1204-2005 | 26 | 0.645 | 0.464 |
| 6 | HB* | 34.76 | 100.81 | 3320 | 347.3 | 1377-2005 | 26 | 0.496 | 0.469 |
| 7 | XES* | 33.73 | 96.25 | 4234 | 275.1 | 1600-2008 | 40 | 0.639 | 0.525 |
| 8 | AS* | 32.65 | 95.68 | 4051 | 386.6 | 1424-2008 | 34 | 0.540 | 0.526 |
| 9 | DZ* | 32.53 | 97.67 | 4018 | 327.5 | 1319-2001 | 31 | 0.510 | 0.470 |
| 10 | DB* | 32.19 | 95.64 | 4192 | 398.7 | 1460-2001 | 29 | 0.667 | 0.391 |
| 11 | BZ* | 31.87 | 96.52 | 3908 | 431.0 | 1314-2001 | 32 | 0.554 | 0.374 |
| 12 | SX* | 31.63 | 94.29 | 3854 | 173.7 | 1494-2004 | 27 | 0.448 | 0.397 |
| 13 | LWQ | 31.59 | 96.57 | 3882 | 275.0 | 1387-2006 | 22 | 0.370 | 0.417 |
| 14 | BB | 31.15 | 95.78 | 4453 | 278.8 | 1394-2006 | 30 | 0.773 | 0.416 |
| 15 | BR* | 31.12 | 93.87 | 4350 | 379.3 | 866-2005 | 31 | 0.554 | 0.620 |
| 16 | MX* | 31.08 | 94.58 | 4144 | 429.9 | 1141-2006 | 24 | 0.620 | 0.428 |
| 17 | CD | 31.08 | 96.97 | 4388 | 309.6 | 1477-2010 | 32 | 0.526 | 0.360 |
| 18 | CY | 31.06 | 97.68 | 4325 | 423.6 | 1252-2006 | 27 | 0.664 | 0.652 |
| 19 | GJ* | 30.75 | 98.69 | 3817 | 318.0 | 1361-2006 | 24 | 0.464 | 0.547 |
| 20 | JL* | 30.60 | 93.60 | 4250 | 245.3 | 1412-2004 | 30 | 0.613 | 0.404 |
| 21 | LL* | 30.58 | 96.18 | 4440 | 311.4 | 1409-2006 | 32 | 0.742 | 0.604 |
| 22 | LZ* | 30.31 | 91.51 | 4233 | 418.0 | 1173-2004 | 30 | 0.578 | 0.613 |
| 23 | BS* | 30.06 | 97.12 | 4282 | 216.3 | 1683-2006 | 29 | 0.535 | 0.264 |
| 24 | MK* | 29.75 | 98.58 | 4050 | 425.0 | 1405-2006 | 28 | 0.553 | 0.693 |
| 25 | LKZ | 28.98 | 90.44 | 4680 | 153.1 | 1739-2002 | 20 | 0.347 | 0.287 |
| 26 | ZJS | 28.86 | 90.53 | 4603 | 101.8 | 1784-2005 | 25 | 0.598 | 0.532 |
| 27 | LD | 28.84 | 93.04 | 3744 | 274.8 | 1486-2013 | 38 | 0.508 | 0.556 |
| 28 | LJ | 28.12 | 90.99 | 4006 | 184.7 | 1715-2004 | 27 | 0.345 | 0.268 |

Site No. is listed in the order from north to south; star sign (*) indicates sites previously reported on journals and updated by our research group; Trees in sites 1~6 are *Juniperus prezwalskii* and the rest are *J. tibetica*.; Mean age of trees (1969) is the averaged ages of trees in 1969 based on the starting year of each samples; Mean R_bt_ refers to mean inter-serial correlation. R_chron-PDSI_ refers to the correlation coefficient between ring-width chronology and May-June PDSI in the interval 1957-2000 A.D..
